# Improving the quality of chronic care through purchasing arrangements in resource-constrained settings: insights from an international Delphi survey

**DOI:** 10.1080/16549716.2025.2518667

**Published:** 2025-07-03

**Authors:** Bruno Meessen, Megumi Rosenberg, Grace Marie V. Ku

**Affiliations:** a Department of Health Economics and Financing, World Health Organization, Geneva, Switzerland; b Centre for Health Development, World Health Organization, Kobe, Japan; c Department of Public Health, Institute of Tropical Medicine, Antwerp, Belgium; d Department of Gerontology, Faculty of Medicine & Pharmacy, Vrije Universiteit Brussel, Brussels, Belgium; e Faculty of Medicine & Surgery, University of Santo Tomas, Manila, Philippines

**Keywords:** Quality of Care for Chronic Conditions, Provider payment, non-communicable diseases, quality of care, health financing, low- and middle-income countries

## Abstract

**Background:**

There are substantial issues with the quality of care (QoC) received by persons living with chronic conditions, particularly in low- and middle-income countries (LMICs). One possible channel to improve QoC is through financing, specifically purchasing arrangements for health services. This has been actively explored in high-income country settings, generating a growing body of scientific knowledge.

**Objective:**

To understand the potential and the constraints of using purchasing arrangements as a way to improve QoC for chronic conditions in resource-constrained settings.

**Methods:**

A Delphi survey was conducted with 49 international participants with content expertise in chronic care management, health financing, or both, and context expertise in resource-constrained settings including in Small Island Developing States or Fragile and Conflict-Affected States, to assess the possible contribution of purchasing arrangements to QoC for chronic conditions with respect to specific types of care providers (e.g. patients and relatives, community health workers, public health centres), decentralized coordination bodies and purchasing agencies in such settings.

**Results:**

There was a high level of consensus among the Delphi panel in favour of considering purchasing arrangements as one of the levers to improve QoC for people living with chronic conditions. Specific directions for action were identified along with their caveats.

**Conclusions:**

The challenge of improving the quality of chronic care in resource-constrained settings is extensive and requires immediate attention. Leveraging purchasing arrangements is one promising channel to strengthen quality chronic care in such settings.

## Paper Context

**• Main findings**: There was consensus among the Delphi panel in favour of considering purchasing arrangements as one of the levers to improve quality of care for people living with a chronic condition in resource-constrained settings.

**• Added knowledge**: Results of this Delphi survey are complementary to evidence already available from high-income countries and take into account constraints and priorities in resource-constrained settings.

**• Global health impact for policy and action**: This work can guide efforts by health authorities and their technical and financial partners on designing, implementing and documenting purchasing instruments to improve quality of chronic care in resource-constrained settings.

## Background

‘Good quality care’, which has been defined as the ‘*right care, at the right time, responding to the service users’ needs and preferences, while minimizing harm and resource waste’* [1], has been a central concern for health professionals, health care users and a much larger set of stakeholders. Quality of care (QoC) is also a global policy goal and has been conceptualized as an intrinsic component of the universal health coverage (UHC) goal adopted by the United Nations [1].

QoC is the result of a large set of determinants. Actors contemplating its improvement can thus consider a wide array of (complementary or not) theories of change. One channel of intervention is through financing, specifically the purchasing arrangements for health services. Purchasing refers to the allocation of pooled funds to health care providers for the delivery of health services on behalf of certain groups or an entire population [2]. Purchasing arrangements are defined as any institutional arrangement allocating and channelling financial resources from a purchaser such as the Ministry of Health or a social health insurance fund to a provider for the provision of health services to reach some health objectives, including UHC. Through the inputs they finance, the information they communicate, the incentive structure they set and the other systemic changes they trigger or enable, they can be powerful levers with direct and indirect impact on the users’ profile and the volume and quality of services. As purchasing arrangements are amenable to revisions, it is key to consider how they can be optimized from a QoC perspective.

To use purchasing arrangements to purposely improve QoC, for instance, through accreditation requirements or pay-for-performance arrangements, is nowadays an established approach in many countries, though with variable results [3]. There is growing literature on the matter, including normative guidance by the World Health Organization (WHO) [4]. In low-and-middle-income countries (LMICs), performance-based financing programmes have been one vehicle for the diffusion of such an approach [5,6], but often with a focus on reproductive, maternal and child health [7]. A recent scoping review focusing on provider payment arrangements geared towards improving quality in chronic care reveals that few experiences and programmes are documented and assessed in LMICs (and those few experiences are in China only) [8].

This is an important gap. Premature morbidity and mortality due to the rapid rise of chronic conditions, especially non-communicable diseases (NCDs), constitute a major challenge in many LMICs [9]. In the years to come, health care facilities will be under greater pressure from this growing burden of chronic conditions. Limited resources set constraints upon service delivery in low-income countries (LICs) and in many middle-income countries (MICs) (e.g. in rural settings and slums). Given the current low level of service coverage [10–12] and limited QoC for chronic conditions [13,14], tailored supportive policy interventions will be needed. These interventions will have to mobilize the different components of the health system, including health financing. This raises the question whether acting on purchasing arrangements – so they better take into account the specificities of chronic care – should be part of the solution.

Recognizing this forthcoming need for policy guidance in LMICs, WHO implemented a programme of work on purchasing quality chronic care [15]. To complement the insights gathered from evidence available mostly in high-income countries (HICs) [8], it was decided to organize a Delphi survey with experts familiar with resource-constrained settings. In this paper, we present the guidance which emerged from the experts as for the potential and the constraints of using purchasing arrangements as a way to improve QoC for chronic conditions in such environments. We review their preferred options per type of health care providers, identify some more systemic issues and put forward directions for action, along with their caveats.

## Methodology

Acting on QoC, regardless of the type of intervention one considers, requires having a good understanding of what really matters for the management of the specific health condition under consideration. Recognizing the absence of a QoC framework tailored to the specificities of chronic conditions considering resource-constrained settings, we carried out a literature review of the conceptual literature and developed specifications of chronic care quality. Results of these preliminary steps are reported in two accompanying papers in this special collection [16,17].

### Delphi survey

Equipped with this advanced understanding of good-quality chronic care, we identified 52 non-WHO professionals with relevant but diverse expertise for a Delphi survey. These individuals were identified through scientific literature and reference searches, professional networks and databases, and snowball sampling (see also 16). The international networks of the authors, GMK and BM, in their own field of work were helpful to contact and recruit experts in chronic care management and health financing, respectively. Particular attention was granted to involve experts working in specific contexts, such as Small Island Developing States or Fragile and Conflict-Affected States. The Delphi survey was organized, in English, in two rounds, via an online application, Mesydel (https://mesydel.com/en).

Forty-nine of the 52 invited individuals (94%) consented and participated in the Delphi survey (46 at round 2), forming the Delphi panel (see acknowledgement section for the full list).

**Map 1.**
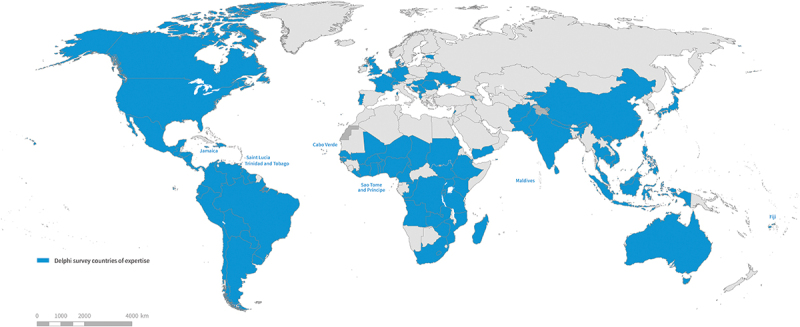
Coverage of country expertise within the Delphi survey panel of 49 participants, 2023^a^.

Demographic characteristics of the panel are provided in Table 1. The panel consisted of experts advanced in their professional career (average age: 49.1 years old), with a majority of them being in a role of advising LMICs’ governments on health policies. It tapped into a diversity of professional capacities (e.g. health policymakers, technical assistants, health care organization or patient group representatives, clinical practitioners, academics) and perspectives (e.g. central government level, facility level, person living with a chronic condition (PwCC)). The geographical and cultural exposures/insights were broad, with panellists residing in 30 countries and with field exposure in 97 countries across the six WHO regions (see Map 1). Exposure to Western Europe and Northern America was not purposely looked for: it often came with experts native or trained in these regions but recruited for their activity in LMICs.

**Table 1.** Demographic characteristics of Delphi respondents (*n* = 49).

Age	Average	49.1 years
	Range	33–65 years
Sex	Female	15
	Male	34
Continent of origin	Africa	10
	Asia	12
	Europe	11
	North America	7
	Oceania	2
	South America	3
	Chose not to disclose	3
	No answer	1
Socio-economic classification of country/ies of ‘expertise’/having knowledge of	Low-income (LIC)	10
	Middle-income (MIC)	10
	High-income (HIC)	3
	All	7
	Both LIC and MIC	13
	Both MIC and HIC	3
	Not applicable	3
Stakeholder characteristics (multiple answers possible)	Clinician/health care provision	15
	Health financing	29
	Policy implementation	19
	Policy formulation	21
	Government adviser	33
	Teacher or researcher in chronic conditions	17
	Teacher or researcher in quality of care	24
	Teacher or researcher in health financing	20
	Informal caregiver of PwCC	5
	PwCC	4
	Civil society representative	2
	Health care organization representative	8
	Patient group representative	4

Small island countries are named because otherwise they may not be visible.

Under round 1, we collected general data on the panellists [7 questions], elicited their views on our prior scoping review findings that underpin the chronic care quality framework [34 questions] [see also 16 and 17], and on how purchasing arrangements could contribute to improving QoC for chronic conditions with respect to specific types of care providers (i.e. patients and relatives, community health workers, public health centres, non-for-profit facilities, private-for-profit clinics, tele-health/m-health providers, hospitals, retail pharmacies), decentralized coordination bodies and purchasing agencies, also with attention to ‘aggregate’ purchasing arrangements (i.e. bundled payment and ceiling on aggregate co-payment) in LMICs [73 questions]. The questionnaire combined a mix of multiple-choice, ranking and open questions. For most providers, the respondent could opt out to answer a question if s/he felt that she lacked information.

The first author of this paper analysed results of round 1, with particular attention to participation rates, the votes expressed by respondents and their many qualitative comments (for a total word count of 24,525 words). The participation rates were variable (Table 2), suggesting that some respondents felt insufficiently informed about some specific types of providers. The votes revealed high consensus within the panel already at round 1 (consensus was defined as 50% or higher agreement among the respondents who had opted in). The review of qualitative comments indicated that divergence was often related to exposure to different contexts. More fundamentally, the qualitative comments were helpful to identify issues or perspectives missing from the original questionnaire. For instance, a comment from a participant led to specifying ‘care integration’ as a point of attention in the second round.

**Table 2.** Number of panellists who preferred not to answer about a specific type of providers.

	Round 1	Round 2
Patients and relatives	Not proposed	1/44
CHWs	16/49	5/45*
Health Centres	6/49	3/44*
Private not-for-profit	26/48	6/44
Private for profit	28/48	5/44
Hospitals	9/48	4/44
Telemedicine	Not proposed	1/44
Private retail pharmacies	Not proposed	6/44
Decentralized coordination bodies	22/47	2/45
Bundled payment and cap on co-payments	12/47 and 20/46	4/44
Purchasing agency	18/48	3/48

*Several statements to vote upon, we report the one with the highest proportion of opt-out.

Based on the data analysis, the first author produced a series of statements synthesising the emerging consensus views on issues with quality of chronic care in resource-constrained settings [5 statements] and the potential role of purchasing arrangements in improving QoC [7 generic statements and 21 statements specific to a type of provider/actor or provider payment mechanism]. The other authors contributed to the verification and refinement of those statements. Then, the second round involved a vote on each of these 33 statements, with the options to decline to respond, to disagree, to agree with no qualifications or to agree with qualifications explained in a comment box (for a total word count of 11,114 words).

## Results

The full results of the Delphi survey (rounds 1 and 2) are available from the Institute of Tropical Medicine Antwerp (see https://www.itg.be/en/research/research-themes/quality-of-care-for-chronic-conditions) [18]. In this paper, we summarize the majority view of the Delphi panel about how each type of provider can contribute to better quality chronic care across the seven pre-identified chronic care quality aims (see [16]) and how purchasing arrangements can support or enhance their contribution to strengthening care quality.

Where we deem it relevant, we provide (i) an indication of the strength of the consensus among those who responded using a score which aggregates the two positive response options (i.e. agree with or without qualifications); and (ii) on the evolution of the proportion of respondents who decided to opt out across the two rounds (see also Table 2, which suggests an overall increased confidence of panellists in their judgement). In our reporting, we also integrate some explanatory information provided to the panelists, which could be helpful for the readers. To reflect the richness of insights collected through the open-ended questions, including dissenting opinions, we have also integrated quotes from the panellists. For several proposed options, the panel was doubtful about their feasibility in resource-constrained settings; where it applies, we make a distinction between LICs and MICs. Several messages with relevance across types of providers were endorsed by the Delphi panel. These are summarized in Box 1.

### Patients and relatives

While self-management and care provided by family and relatives are key for chronic condition management [17], the panel was not favourable to reward PwCC for successfully taking care of themselves, for instance, a financial reward for successful smoking cessation [at round 1, only 15% supported such schemes]. If any compensation is paid to PwCC, the consensus, reached at round 2 [91% of agreement], was that the focus should be on improving accessibility by reducing financial barriers to accessing care or taking appropriate health-seeking behaviour, including testing, for instance through a voucher, a conditional cash transfer or a reimbursement of costs such as for transport. The panel considered that it would be fair to offer financial support to family members taking care of PwCC on a limited basis but observed this may require quite advanced safety net mechanisms. Ensuring that households with a PwCC are entitled to social welfare support, for instance, through the recognition that having a chronic condition is an extra source of economic vulnerability, was perceived as the most practical step to take. In their comments, several panelists remarked that caution is warranted in this respect; as one of them put it: ‘*in part, this is a question of relative cost-effectiveness given the scarce resources*’.

### Community health workers

The panel agreed that community health workers (CHW) can contribute to quality chronic care, especially in the dimensions of accessibility and efficiency. If CHW are themselves PwCC, their personal experience can be an asset to support continuity, for example, by encouraging their peers to have a good observance of the treatment plan, and to support person-centredness, for example, by offering credible tips for adapting their daily life.

Different options of payment models for CHW were considered in round 1 [66% of support for payment to CHW], none stood out. The main recommendation is that the overall contractual arrangement ensures that prerequisites for QoC are fulfiled. These include, among others, a well-defined scope of work, prior training on assigned tasks, continuous training on other skills, funding for running costs, provision with screening and health promotion materials, and supervision. In turn, they would be required to report activities [91% of agreement].

If the context is favourable, such as the country has a positive experience with similar arrangements with other levels of care or other health needs, a pay-for-performance arrangement can be considered [74% of agreement within the panel]. It could, for instance, reward the CHW who has identified a person at risk and persuaded her to take a screening test and come to the health centre for confirmatory diagnosis. However, as noted by a panelist: ‘*this is unlikely to be sufficient given the steep drop in the care cascade. I would consider this as a partial intervention*’.^1^1The cascade of care is an analytical framework to evaluate public health interventions; it constructs models of the proportion of people completing sequential steps of care for a given condition or disease. For an example, see our reference 37.

### Public health centres

Strategic purchasing is also about deciding from which providers to purchase [2]. The panel suggested assigning a central role to public health centres in the management of chronic conditions, at least in contexts where they constitute the backbone of first line services [98% of agreement]. They are seen as particularly key in ensuring accessibility, efficiency, equity, person-centredness and continuity. There may be few alternatives available in rural areas.

With respect to public health centres, an upfront concern of our panellists was ensuring that all key conditions for QoC to be delivered are fulfiled. In many LMICs, this may require a substantial technical upgrade of public health facilities, which will require governments to allocate them more resources, but with full cognizance that care improvement will require more than mere injection of inputs. Commitment to improvement processes at facility level will be key.

A majority of the panel recommended that the allocation formula of the extra financial resources to the public health centres considers the volume of services [83% of endorsement at round 1], the quality of services [83% of endorsement at round 1] and the socio-economic conditions of the served population [60% of endorsement at round 1].

Already at round 1, there was a strong preference within the panel for some form of capitation model to take patient or work volume into account (78% of endorsement across the three options proposed). This position is consistent with the recent Lancet Global Health Commission on financing for primary health care which recommends blended payment with capitation as the core payment mechanism for primary care [19]. Round 2 consolidated the consensus [86% of agreement] that capitation models can indeed be a way to take volume of work and socio-economic conditions of the served population into account, without encouraging over-prescription and cost-escalation. The panel acknowledged the potential of capitation with empanelment [84% of agreement]. Under a capitation with empanelment system, patients choose and register at the health centre they plan to use if they need health services. Health centres receive a prospective fixed payment per registered patient at a given frequency, such as each quarter. The payment is kept by the facility even if the patient does not come to the health facility during the covered period. This creates an incentive for the health centre to keep the patient healthy. This was viewed by the panel to have a high potential for better management of chronic conditions, given that it would strengthen the relationship between the PwCC and her/his health centre and enable longitudinal follow-up by providers. This would be beneficial for person-centredness and continuity of care. However, as noted by one panellist, *‘Contexts with displacement of populations (for whichever reason: conflict, climate, economic, seasonal,…) would need different provisions to ensure displaced or migrating or fleeing PwCC still get access*’. Organizing a capitation system not limited to PwCC might be the ideal goal, but its political feasibility may be limited in many countries. Encouraging first the empanelment of people who need longitudinal follow-up, like PwCC, could be a reasonable first step for piloting this provider payment model.

As mentioned above, to enable public health centres to provide quality chronic care, a full set of measures may be required. For the panellists [91% of agreement], the empanelment has another potential advantage in that it provides an extra reason to introduce, where it is still absent, the individual data system allowing longitudinal follow-up of the patient. Ideally, this would take the format of an electronic health record (EHR) system allowing recording by different providers and facilities, or at least an electronic medical record system maintained at the level of the health centre. Data protection should be a priority and will entail extra recurrent costs. Ideally, for integration purpose, the EHR should tap the unique identifier from the civil registration system, if this exists, rather than a sector-specific identifier. However, as one of the panellists noted, digitalization is not a magic bullet either: ‘*While the electronic data system can help support patient monitoring/follow-up, digital technologies are only a means to delivering continuous care. Continuous care requires other actions such as support (leadership, managerial and financial) to health workers to innovate and deliver this type of care’.*

It was mentioned that digitalization also comes with its own challenges: *‘Digital solutions can be very efficient and time-saving. They also often offer a way to improve follow up of patients through availability of all information of patients. However, they have the potential to be problematic in terms of use, maintenance (and troubleshooting) and data protection. This last one can be of more important in LMIC prone to conflicts. Digital solutions should also not undermine the self-management and empowerment of patients, by reducing the attention to give patients their health information in hard copy’.*

Regarding other components of the blended provider payment, a fair number of panellists were supportive of introducing a pay-for-performance component [79% of agreement]. In places where there is an effective pay-for-performance programme for health centres, adding some chronic care indicators to the performance checklist is an option. A possible first step could be to reward actions which should be done already. One prerequisite to move in this direction may be the establishment of a good individual digital data system. A panellist also recommended that the pay-for-performance covers ‘*health worker competency (knowledge, skill and ability) to deliver these interventions*’.

Comments by panellists suggest that for finding the blend appropriate to a specific context, a focus on the patient’s experience and outcomes will be required and piloting will be key.

### Private facilities

The panel was also asked about conditions to be put on private not-for-profit and private for-profit providers before any reimbursement by some pooled fund managed by the public authorities, such as social health insurance. This is particularly key in contexts where these providers are, de jure or de facto, the favoured source of care for PwCC or patients in general. *‘In [country name], over 90% of primary providers are private. A focus on public centers would be tantamount to reducing access to care across the board’*. The panel recommended the purchasing agency to be strict on both the eligibility criteria and the mechanisms to ensure accountability. As put by a panellist: *‘I still think public-private partnership, mainly through purchasing arrangements, has a strong potential in a context of relatively weak governance. A contract with selected/assessed/accredited private providers by a third-party purchaser is an entry point for regulations, access to their data and quality assurance’.*

For specialized private not-for-profit facilities, the following orientations obtained backing at round 2 [82% of agreement]. Regarding contracting and payment arrangements, several options were deemed possible, for instance a multi-year block grant contract with obligation for the facilities to report frequently, including a narrative report, or different variations of a capitation system. Monitoring of performance should be in place and a pay-for-performance can be considered. Ideally, the latter should focus on outcomes, namely that the disease is under control. Such an advanced payment system, however, requires a reliable electronic medical record system, and supportive leadership. If the centre is specialized in chronic conditions, the purchasing agency should make sure that the arrangement clearly identifies the systemic role assigned to the centre and ensure its commitment to achieve optimum integration with the rest of the health system, including public health centres and hospitals. Adoption of the EHR system used by public facilities, or at least interoperability, could be put as a requirement, but integration should also be sought on referral service delivery and many other aspects including supervision, peer exchange, training and research. This is one of the providers for which there was the highest number of panellists who preferred not to answer [54% at round 1, still 14% at round 2], likely due to lack in first-hand experience, even though this type of care provider was represented in the panel.

The panel recommended being conservative with enrolment of private for-profit clinics under the pooled funding with the objective to integrate them more effectively into the whole health system. Their eligibility to reimbursement from the pooled fund should be conditioned on advanced accountability in terms of transparency and data reporting, and ideally accreditation [75% of agreement]. The panel expects private for-profit clinics will request a fee-for-service model. As such, offering other options with more desirable effects on QoC should be explored. Capitation could be one of these options [80% of agreement]. Noteworthy, private for-profit providers were the category of providers about which the panellists were least forthcoming with recommendations [58% opted out at round 1; at round 2, 11% chose not to answer]. This may reflect the diversity in the role that the private sector plays in chronic care across settings but also the relative ignorance which prevails in the community of experts on how to handle private providers.

The panel supported the assessment that in many countries, requesting private providers to comply with prescription protocol for chronic illnesses would be very beneficial for the PwCC attending their services [82% of agreement]. Gains in terms of effectiveness, efficiency, continuity, person-centredness, accessibility, as well as safety are anticipated.

In general, the panel recommended enrolling private practitioners in a pooled fund, provided that the purchasing agency commits to analysing prescription behaviours with a suitable data system, analytical capacities and enforcement measures (see also sections on private retail pharmacies and purchasing agencies). One reason for that is, nowadays, some of the most expensive medicines relate to NCDs. If they are reimbursed by the pooled fund without proper checks, they can quickly weigh heavily on the whole budget.

### Hospitals

As for hospitals, a key finding of round 1 was the very strong endorsement for an allocation based on volume and types of services delivered [89% of endorsement], along with majority support for paying for quality [70% of endorsement] and for some measures of the local socio-economic reality, such as a higher payment in remote or poverty-stricken areas [54% of endorsement].

At round 2, we opted to invite panellists to position themselves on some general orientations acknowledging the role of hospitals in NCD care. Panellists approved [86% of agreement] the following key orientations.

Purchasing arrangements should ensure that hospitals complement primary care providers and thus support a focus of hospitals on investigation and diagnosis, intervention, inpatient care and appropriate discharge, coordination and restoration for the best continuity of care.

When linking the payment to the volume of services, the formula should have the sophistication that the health system can afford. Advanced case-based payment organized around a long list of diagnosis-related groups (DRGs) was not seen as a realistic option for LICs. More simplified systems could be explored. Provider payment reforms should be incremental and it should be assumed that chronic conditions will not be the drivers of hospital payment reforms in those contexts.

Across settings, provider payment reforms for hospitals should strive for the best integration of hospitals into the whole health system. Their contribution to the primary health care agenda lies in focusing on what first-line services cannot and should not do. They should also play their part in strengthening the continuity of care, including by counter-referral notifications, such as for patients who did not know that they had a chronic condition.

Given their multiple missions, financing of hospitals in most LMICs will remain shaped by a set of mixed payment mechanisms. For instance, the 24/7 availability of services, which has a lot of relevance for PwCC as well, deserves attention. During the COVID-19 crisis response and the subsequent massive cancellation of elective surgeries and non-vital interventions, this capacity was jeopardized in some hospitals relying on actual volume of activities for their income.

### Telemedicine

The panel welcomed developments in LMICs for patient-to-provider teleconsultation and professional-to-professional tele-expertise. The benefits of teleconsultation for the PwCC could be in terms of accessibility, for example, for patients with mobility constraints to visit their first-line services, and continuity of care, possibly to have a medical follow-up and a renewal of prescription without travel or face-to-face contact, as during the COVID lock-down. The benefits of tele-expertise between a first-line provider and a specialist medical doctor, or another team member, are in terms of indirect accessibility to advanced services, for instance, for PwCC living in a small island, effectiveness such as reliability of the diagnosis and appropriateness of the prescription, and efficiency. In many LMICs, just like in HICs, there is still a need to pilot, document and fine-tune several aspects of these solutions. Pooled funds would need to learn how to compensate providers doing teleconsultation and specialist doctors advising first-line doctors [95% of agreement].

### Private retail pharmacies

In many LMICs, medicines bought at private retail pharmacies are not yet covered by pooled funds and are paid for by patients themselves. When the panel was asked whether countries should set up purchasing arrangements ensuring coverage by the pooled fund of medicines for chronic illnesses, they were supportive of piloting or scaling up such a solution [agreement: 86%], but with several caveats. Their assessment is that retail pharmacies are insufficiently regulated in most LMICs, and in many of them, unregulatable in the current state of affairs – this is a major constraint for integrating them under the pooled fund. For countries taking this route, the ambition should be that the arrangement covers all the medicines on the national essential drug list, with clear reimbursement rate, through ideally an e-prescription system ensuring that the subsidy is directly charged to the pooled fund and not pre-paid by the patient. Starting with medicines for chronic conditions could be a smart approach. This is a group of patients for whom an advanced follow-up system is anyway required [see above and 16]. Thus, the data system might be compatible with what is required by the subsidized medicine scheme. These are also medicines which can be costly for the patients, and for the whole society. Limiting the scheme to generics and securing enough monitoring control will be key. Private retail pharmacies were another category of providers for which there was a high number of panellists who preferred not to answer [14%].

### Support decentralized coordination bodies

At round 1, many panellists preferred to opt out on the questions relating to decentralized coordination bodies [47%]. At round 2, participation was much higher [only 2% preferred not to express themselves] and there was close to perfect consensus on the proposed statement [96% of agreement]. Decentralized coordination bodies were seen as key to rolling out delivery models supportive of better management of chronic care in LMICs. Although required capacities at their level were not seen as specific to chronic conditions, the feedback was that in most LMICs, coordination bodies like district health offices will have to build a stronger understanding and expertise in chronic illnesses. They will have to develop their capacities in their role of coordinating providers, including towards private facilities and social affairs actors, a weak point in many settings and learning especially from the routine information system after its upgrade with the digitalization of patient files. The panel did not call for any major change in their financing model. There was consensus on the proposition that setting a link with some measures of quality might be welcomed in some settings.

### Payment models covering several providers

The panel was also asked to consider purchasing and benefits arrangements that ‘aggregate’ different types of providers. Two models were presented in the survey. Bundled payment means grouping together several components of health care delivered by several providers for a specific intervention and paying for the whole ‘bundle’ together, such as across disciplines and care levels. It provides incentives for the integration of care and patient-centred collaboration and coordination across providers. Bundling is based on the expected costs of a patient’s case, an episode or care over a specified period, whereby payment is made to a provider network. The other strategy proposed to the panel was to set a cap to the aggregated co-payments paid by the patient, possibly across providers, for a given period. Above a given ceiling, possibly set according to the household income, patients are exempted from co-payment or all co-payments are reimbursed. This solution allows to protect PwCC from catastrophic health care expenditure and poverty and secures good continuity of care.

At round 1, many panellists preferred not to answer to the related questions [respectively, 26% and 43%]. Several also expressed their doubts or even their opposition: ‘*I think bundling is a flawed strategy in the context of multimorbidity which is the rule, not the exception, in our system. Bundling privileges certain conditions and diminishes the importance of others and is often conceptualized in the absence of lived experience of patients’*.

At round 2, there was a higher participation [9% opted out] and panellists supported the two models [agreement: 89%], but with caveats. As put by a panellist: ‘*The more advanced system of payment (though can be ideal), the higher capacity of both human resource, information and communication technologies and system is needed, and also the higher cost incurred. So, I prefer a relatively simple method for developing countries’.*

Panellists agreed that theoretically, the bundled payment model may positively contribute to quality dimensions such as effectiveness, efficiency, continuity and person-centredness, as it creates incentives for better coordination between the different health care providers but also for investing in teaching PwCC how to self-manage their condition. A panellist flagged that shared savings, in which any surplus is appropriated by the network, is expected to incentivize a shift of care and resources from hospitals to primary care. The experts also believe that a ceiling on aggregate co-payments can positively contribute to quality dimensions such as equity and financial accessibility and thus improve continuity and effectiveness of the care management. Our experts, however, recommend realism on the feasibility of these two aggregative models of payment. Their assessment is that they are not feasible options in most LICs. These solutions are too demanding in terms of coordination, data system and financial management to be implemented in resource-constrained settings. Our experts were more positive on the applicability of these two solutions in MICs (still, several experts marked scepticism for their own MIC). They recommend piloting and even scaling up, but only once a proof of concept is available. For the bundled payment model, they recommend not to initiate it as a stand-alone solution but rather as part of a broader effort towards health care integration.

### Purchasing agencies

Leveraging purchasing arrangements for quality chronic care will require capable purchasing agencies. At round 1, 37% of panellists preferred not to contribute to the related question; 6% opted out at round 2. At round 2, there was strong support for the following general assessment and orientation [92% of agreement].

The panel considers that in most LMICs, capacities of purchasing agencies must be strengthened. Most of them fail to exploit the wealth of data generated by payment systems and even more, data from other sources. They have been capacitated to process claims and payments, not to generate intelligence for the health system. Often, they do not see themselves as having a role in influencing the QoC.

Actually, several of the paths for action listed above do require that countries strengthen their purchasing agencies. This can be done step-by-step on a learning mode, with the support of the broader knowledge ecosystem. The latter will be particularly key for securing efficiency at system level, thanks to health technology assessment. Determination of a national benefit package can also contribute to equity and enhance the possibility to enforce guidelines to the benefit of effectiveness and safety, though one respondent casted doubts on gains achieved so far through benefit package design.

**Box 1:** Consensus views on quality chronic care and purchasing arrangements that apply across different provider types.

Acknowledge cross-cutting issues such as equity [95%] or the social determinants of chronic conditions [comment received at round 2] and integrate action within broader societal transformation such as the UHC agenda Embrace a multidimensional perspective of quality chronic care, with person-centredness and continuity as the two dimensions which are the most specific to chronic condition [100%] Adopt integration as a key ‘organizing principle’ for service delivery and beyond, and accompany the reshuffling of service delivery accordingly [98%] Act structurally on quality of chronic care shortcomings, with a health system perspective on actual bottlenecks to quality of chronic care [98%] Assess whether the default purchasing arrangements give proper consideration to the specificities of managing chronic illnesses; if it is not the case, consider modifying them [100%], but ensure that action on purchasing arrangements is embedded into the broader health financing perspective [98%] Use purchasing arrangements for what they can achieve best, especially accessibility [98%], effectiveness [93%], continuity and person-centredness [95%] Value the various mechanisms activated by the theory of change of purchasing, with attention to undesirable effects [95%] Tailor the purchasing arrangements to the (evolving) context and invest in learning [100%]Between brackets: agreement rate for related statements at round 2

## Discussion

The international panel of experts recognized the extensiveness of the challenge of improving quality chronic care in resource-constrained settings and the need to act. They saw promise in purchasing arrangements as one of the channels to improve quality chronic care. They have reviewed a large set of provider payment options for different types of providers and helped us to assess their relevance and feasibility in resource-constrained settings. In this discussion section, we highlight some possible implications of the findings for actors in a position to advance this policy and learning agenda, including at global level, and relate some results to the broader literature.

The Delphi panellists stressed the requirement that any undertaking to improve QoC for chronic conditions through purchasing arrangements is integrated within broader policy efforts, including those related to health care delivery, health financing for UHC and health system strengthening in general and even wider societal transformations to fight NCD determinants (Box 1). Given the focus of this journal issue, we focus on the link between purchasing arrangements and service delivery.

The results of the Delphi survey support the notion that there is value in considering purchasing arrangements as a tool for improving QoC for chronic conditions. Chronic conditions, by definition, are not managed at one point of time at one place. There is a long evolution with the condition and an accompanying ‘journey’ with health care [16]. This raises the key question of the organization of interventions and care across time and providers, often with a backdrop of multiple morbidities. As developed elsewhere in this Special Collection [16,17], this creates specific challenges for the delivery of quality care, especially for the core dimensions of continuity of care and person-centredness. In many HICs, this has led health system stewards to engage in a difficult reshuffle of the health care system. Several of them have been leveraging purchasing arrangements to support their reform of delivery models and align providers on new objectives such as ‘integrated care’, through payment models like pay-for-performance, blended capitation or bundled payments. This movement has been accompanied by a growing literature and some emerging lessons for these settings [8,20,21].

This Delphi panel was organized to fill a gap in knowledge about how this might apply to LMICs, but also to raise attention to the necessity in LMICs to act jointly on models of care and purchasing arrangements. In these contexts, the challenge is the current backdrop of under-funding of NCD care, and its consequence, the under-provision by public facilities [10–12], as noted by the panellists.

The panel has proposed ways forward. It has tried to be comprehensive in its understanding of issues and their underlying sources and to consider a large scope of solutions. For instance, beyond the adoption of a multidimensional approach to QoC, the panel has also paid attention to financial protection (cf. the proposition to put a cap on aggregate co-payments). There is indeed a growing literature showing that chronic illnesses are an important driver of catastrophic health care expenditure [22,23]. From a health financing perspective, the burden of NCDs will quickly be compelling, for households but also for nations. The fact that NCDs also affect wealthier groups means that rationing by public under-provision will not contain costs much. It will just allow more determination by market mechanisms, and thus an even larger role of the private sector and for those without an insurance coverage, higher out-of-pocket expenditure [24]. Lack of proactivity fuels dangerous trends. For instance, in Vietnam, according to a recent study, around 5,000,000 hospitalizations per year could be avoided. Among those preventable hospital admissions, close to 50% are related to chronic conditions [25]. The uncapped reimbursement of hospitals along a fee-for-service model is part of the explanation of this over-use of hospital services. Such examples indicate the need for a much stronger action by health system stewards, including a reconsideration of the key role of primary care, a better articulation between different levels of care and most probably a revision of the purchasing arrangements. Tackling NCDs calls for comprehensive systemic action, across building blocks and knowledge silos, a disposition and an ability one still sees too little nowadays in global health or at country level.

Some MICs, especially those confronted with an ageing population, demonstrate willingness to act simultaneously on several levers, including by reforming models of care and adopting purchasing arrangements incentivizing greater integration between providers, but they are too few [26,27]. Acknowledging that prerequisites for sophisticated multi-provider payment models are not yet fulfiled in most LMICs, our panel recommends models still focused on individual health facilities and on securing basic capacities, including availability of qualified staff and medicines. How to remunerate health service providers to ensure that they appropriately take care of PwCC should be a priority area for empirical research in LMICs. There is much too little evidence, across types of providers [28–32]. To a fair extent, this reflects the overall too low priority given to NCDs by governments and the donor community. This very limited body of evidence leaves many questions opened, including on the how.

A limitation of this Delphi survey is that it did not explicitly address the question of the overall health financing system which would be the most conducive to quality chronic care. According to the panel, quality chronic care is first a matter of securing core funding for the activities and key inputs. This suggests that some improvement could already be achieved under an old-style budget line-item system to the extent that it demonstrates an ability to pay regularly for items such as salaries and medicines and to support the deployment of an advanced primary health care strategy. This is consistent with achievements done, for instance, by Costa Rica [33]. With this Delphi panel, we have actually mainly explored purchasing mechanisms which are more data-based, in consistency with normative recommendations by WHO to make purchasing more strategic [2]. For primary care facilities, the Delphi panel expressed support for the proposition recently put forward by the Lancet Global Health Commission on health financing for primary health care [19] in favour of context-specific blended payment built on capitation. But even for such models, we need more documented experience in LICs to identify desirable features of the capitation (e.g. any risk-adjustment for chronic conditions or age) or of the blend (e.g. the nature of any pay-for-performance component), assess acceptability by private providers, confirm feasibility in resource-constrained settings and demonstrate the benefits for PwCC.

Another limitation of the Delphi survey is that it did not review how countries could institutionalize such provider payment models under domestic pooled funding. It seems that a growing number of LMICs want to go towards UHC through national health insurance. This could be the avenue for aligning private providers on UHC, including quality chronic care. Paying more attention to appropriate engagement of private providers is much needed [34], as there are indications that private providers cater to a big share of the NCD care ‘market’, not without consequence for the spending incurred by households [24,35]. However, it is unclear whether the social health insurance agencies being set up in many LMICs have fully recognized the height of the challenge at this level. Many reimburse health facilities along a fee-for-service payment model, which is not the most conducive one for quality chronic care. Their investment in health promotion and prevention interventions, which would be the most cost-effective approach to control costs, seems also too limited [36].

The panel gives some direction on efforts to be done as for capacity building and systemic learning. Some provider payment propositions supported by the panel are – and this is of course welcomed – not so specific to chronic conditions. The main recommendation to researchers working on such arrangements might then be to extend their attention to outcomes for PwCC. Some propositions entail a shift away from existing practices and capacities. A key requirement will be to check feasibility of new ideas in specific contexts. Given the many unknowns, good systemic diagnoses, for instance, with cascade of care analyses [37], participatory design, piloting, monitoring effects and iterative learning will be key. The learning needs are huge at country, regional and global levels. Good documentation of pilot projects and their further scale-up will thus be welcomed, given the limited evidence available today at global level. This should be part of broader efforts in favour of strategic purchasing. In countries already more advanced, there is still a need for working on coordination and accountability mechanisms, investing in data collection and developing analytical capacities, among others [38]. This suggests that international partners, especially those funding activities at operational level, and regional bodies could have a catalytic role to play for the best development of this global learning agenda.

This work on a still-nascent learning agenda has the obvious limitations of a Delphi survey. The profile of the facilitators and the composition of the panel may have influenced some orientations. We have also reported that some respondents sometimes opted for not answering to some questions because of their limited knowledge on a specific type of providers or provider payment mechanism. A Delphi survey reports a facilitated consensus of informed opinions, not a synthesis of empirical studies. The propositions are intended to be instructive, not prescriptive. Context will matter a lot. The fact that in many LMICs, acting on purchasing arrangements may be part of the solution for a higher quality of chronic care should not distract the attention from all the complementary and broader efforts for improving QoC [1].

## Conclusion

The Delphi panel supported the view that purchasing arrangements can be one of the levers to improve quality chronic care in resource-constrained settings. Acting upon them should be done with care, and as part of a broader effort to build financing systems for universal health coverage, transform health care delivery models for the new reality of chronic conditions and improve QoC in general.

In many countries, some substantial changes are needed [39]. Progress will require, among other things, much more collaboration between communities of expertise which have insufficiently coordinated their work so far. Health financing experts of LMICs would benefit from developing a more advanced understanding of chronic care and of the lived experience of PwCC [40]. Similarly, NCD experts should pay more attention to the leverage effect of purchasing arrangements instead of focusing on funding only. Their collaboration will also be key for addressing issues beyond QoC *stricto sensu*, such as the fact that chronic illnesses might be an important driver of catastrophic health care expenditure in their context. The inclusive and participatory approach adopted for this work could be adopted at country level. Convergence between experts in chronic conditions (including PwCCs), in QoC and in health financing will be a real asset to handle political constraints and other challenges that many countries will face in the much needed reform of their health system.
